# Research on Rotary Magnetorheological Finishing of the Inner Surface of Stainless Steel Slender Tubes

**DOI:** 10.3390/mi16070763

**Published:** 2025-06-29

**Authors:** Zhaoyang Luo, Chunya Wu, Ziyuan Jin, Bing Guo, Shengdong Gao, Kailei Luo, Huiyong Liu, Mingjun Chen

**Affiliations:** 1 School of Mechatronics Engineering, Harbin Institute of Technology, Harbin 150001, China; 23s008096@stu.hit.edu.cn (Z.L.); jin_ziyuan@163.com (Z.J.); guobing@hit.edu.cn (B.G.); sdgao@hit.edu.cn (S.G.); 2 State Key Laboratory of Robotics and System, Harbin Institute of Technology, Harbin 150001, China; chenmj@hit.edu.cn; 3 Dongguan Ju Rui Electrical Technology Co., Ltd., Dongguan 523400, China; luokailei@juray-elec.com (K.L.); liuhuiyong@juray-elec.com (H.L.)

**Keywords:** magnetorheological processing, stainless steel tube, internal surface polishing

## Abstract

316L stainless steel slender tubes with smooth inner surfaces play an important role in fields such as aerospace and medical testing. In order to solve the challenge of difficult machining of their inner surfaces, this paper introduces a novel rotary magnetorheological finishing (RMRF) method specifically designed for processing the inner surfaces of slender tubes. This method does not require frequent replacement of the polishing medium during the processing, which helps to simplify the processing technology. By combining the rotational motion of a magnetic field with the linear reciprocating movement of the workpiece, uniform material removal on the inner surfaces of 316L stainless steel tubes was achieved. Initially, a finite element model coupling the magnetic and flow fields was developed to investigate the flow behavior of the MPF under a rotating magnetic field, to examine the theoretical feasibility of the proposed polishing principle. Subsequently, experimental validation was performed using a custom-designed polishing apparatus. Through processing experiments, with surface quality designated as the index, the influences of key parameters such as the volume content and sizes of carbonyl iron particles and abrasive particles in the MPF were comprehensively evaluated, and the composition and ratio of the MPF were optimized. Based on the optimized formulation, the optimal processing time was established, reducing the inner surface roughness from an initial Sa of approximately 320 nm to 28 nm, and effectively eliminating the original defects.

## 1. Introduction

Stainless steel slender tubes of different sizes play a key role in a variety of fields, including aerospace, automotive, medical devices, and fluid transfer systems, such as injectors, exhausts, sampling needles, and piping, due to their smooth surface, excellent corrosion resistance, and superior mechanical properties. Despite the divergent application scenarios in these fields, there is a general requirement for high internal surface quality, with the objective of minimizing friction, reducing fluid residue, and enhancing conveyance stability. Typically, slender tubes are manufactured through a drawing process, which often results in poor internal surface quality. The confined and elongated geometry of these tubes present a significant challenge for conventional internal surface polishing techniques to achieve an effective finish [1].

To achieve ultra-precision polishing of the inner surfaces of slender tubes, a variety of non-traditional polishing techniques have emerged in recent years. These techniques include chemical mechanical polishing (CMP) [2], abrasive flow machining (AFM) [3], magnetic abrasive finishing (MAF) [4,5,6], and other magnetic field-assisted methods, such as magnetorheological abrasive flow polishing (MRAFF) [7]. CMP enhances surface smoothness by selectively dissolving microscopic protrusions on metal surfaces, thereby achieving full-surface polishing of complex geometries. However, the chemical fluid employed in CMP often contains corrosive and toxic substances, which can result in environmental pollution [8,9] and pose health risks to operators [10].

AFM utilizes a semi-fluid medium containing fine abrasive particles under high pressure to precisely polish the surfaces of workpieces. This technique is widely employed for processing internal cavities, curved holes, and other areas that are difficult to be accessed using traditional polishing methods. For example, Singh et al. [11] applied AFM to polish the inner surface of 316L stainless steel microholes with a diameter of 850 μm, reducing the surface roughness from 1.4 μm to 150 nm. Similarly, Sharma et al. [12] developed an ultrasonic-assisted abrasive flow machining (UAAFM) processing method that incorporates ultrasonic vibration into traditional AFM, forcing the abrasive particles to perpendicular to the work surface and exert an additional radial force on surface defects to significantly enhance efficiency. Nevertheless, while the incorporation of ultrasonic vibration enhances polishing efficiency, it substantially increases equipment complexity and introduces a multitude of process parameters that are difficult to optimize. Moreover, this technique imposes stringent requirements on metal hardness, toughness, and tube wall thickness, thereby limiting its applicability. Although scholars have made many improvements to AFM, it still encounters challenges in faces controlling the polishing force. In the context of slender tubes, the limited accessibility of abrasive particles [13] and the non-uniform pressure distribution of the polishing medium critically affect surface uniformity, often resulting in over-polishing phenomena near the inlet of the flow channel [14].

MAF utilizes magnetic forces to aggregate ferromagnetic abrasives into a flexible magnetic brush, which exerts pressure on the workpiece surface. As the magnetic field gradient and direction change, the magnetic brush moves along the direction of the magnetic field gradient, creating relative motion with the workpiece surface that achieves material removal [15]. Cheng et al. [16] employed magnetic abrasives with a silicone gel base to polish a 304 stainless steel tube (80 mm in length and 32 mm in diameter), reducing the value of surface roughness to lower than 0.06 μm; however, the high viscosity of the polishing medium limited its flowability, thereby limiting the polishing efficiency and affecting the surface uniformity.

Zhang et al. [17] developed a novel magnetic polishing tool by coating spherical magnets with abrasive particles, enabling localized inner surface finishing of a 316L stainless steel tube (length: 100 mm; inner diameter: 15 mm) and reducing the surface roughness to Ra 0.258 μm. Yamaguchi et al. [18] investigated the effects of magnetic field strength and ferromagnetic size on polishing performance and proposed a two-step polishing method in which workpieces were polished sequentially using magnetic abrasives containing 330 μm and 150 μm ferromagnetic particles, respectively. The inner wall of a 304 stainless steel elbow with a diameter of 8 mm was polished to reduce the roughness value to 0.2 μm, but it was still very difficult to maintain the uniformity of the magnetic field on the surface to be machined. An uneven distribution of the magnetic field results in inconsistent removal of different positions in the tube, local over-polishing, or untreated areas.

In contrast, magnetorheological finishing (MRF) enables precise control of polishing force based on the rheological properties of magnetorheological polishing fluid (MPF) under the influence of magnetic field [19,20,21,22]. Early applications of MRF concentrated on the ultra-precision finishing of optical components, achieving exceptionally smooth surfaces with roughness value below 10 nm [23]. More recently, this technology has been extended to the internal surface finishing of workpieces. Zhang et al. [24] developed a novel magnetically driven polishing tool to polish the inner surface of a tube by placing a spherical magnet inside. The surface roughness value of a 316L stainless steel tube (100 mm length and 11 mm inner diameter) was reduced to 0.08 μm. However, the spherical magnets in the tube were difficult to control effectively and accurately, resulting in somewhat random polishing trajectories that compromised surface uniformity. Song et al. [25] designed a magnetorheological finishing device with a composite magnetic field by combining the rotation of the workpiece with the reciprocating linear motion of the polishing tool and applied this device to the polishing of a titanium alloy tube (100 mm in length and 18 mm in inner diameter), reducing surface roughness value by 0.3 μm. However, the problem of uneven distribution in polishing media still exists, which is not conducive to the improvement in surface uniformity. Li et al. [26] combined multi-pole magnetorheological polishing with shear thickening polishing techniques to polish a slender aluminum alloy tube with a 6 mm outer diameter, reducing the roughness from 480 nm to 155 nm, and achieving a smooth surface free of micro-convex peaks and deep scratches after 50 min of polishing, but the polishing fluid needed to be updated frequently, which seriously affected the processing efficiency. Bahiuddin et al. [27] creatively proposed a roughness prediction model based on machine learning. By combining the initial surface conditions and key process parameters, they accurately predicted the influence law of the content of polyamide 6 in MPF on surface roughness. Yang et al. [28] optimized the process flow of the magnetorheological polishing technology for microcrystalline glass. They obtained the optimal parameter combination through single-factor experiments and response surface experiments. By conducting multi-stage polishing, the polishing efficiency was increased by 26.7%.

This paper proposes a novel rotary magnetorheological finishing (RMRF) method that integrates the rotational motion of the magnetic field with the reciprocating movement of the workpiece to achieve uniform polishing of the inner surface of 316L stainless steel slender tubes, and the polishing medium can be updated in real time during the processing. Based on finite element simulation and processing experiments, the feasibility of the RMRF method is verified. Through a series of polishing experiments, the effects of the main components and parameters of the MPF on the surface roughness, including the sizes and volume content of carbonyl iron particles (CIPs) and abrasive particles, were obtained, and the optimal processing time was determined.

## 2. Principle and Methods

### 2.1. Material Removal Principle

The MPF is a critical part of the magnetorheological finishing process, and its properties directly influence both processing quality and efficiency, which are essential for achieving effective polishing. Typically, this fluid consists of magnetic particles, abrasive particles, a base fluid, dispersants, and other additives. Magnetic particles and abrasive particles are the main components in MPF.

#### 2.1.1. Influence of Magnetic Particles

The rheological effect of MPF, which transforms into a solid state under the influence of a magnetic field, is realized through magnetic particles that form magnetic dipoles and subsequently form magnetic chain structures through their interaction. Therefore, the content and size of the magnetic particles are the main factors affecting the shear yield strength of the MPF, which directly influences the material removal ability. The most typical common formula for the shear yield stress of MPFs is proposed by Kordonski et al. based on the attraction of two magnetic particles in a magnetic field [29]:

(1) $$\tau_{0}=\frac{9\varphi MH^{2}\beta f}{2\pi }$$

(2) $$\beta =\frac{\mu_{i}-\mu }{\mu_{i}+2\mu }$$

(3) $$\varphi f=\frac{a^{4}}{r^{4}}\left[\left(2f_{1}+2f_{2}\right)cos\theta sin\theta -f_{3}{sin}^{3}\theta \right]$$

φ  is the volume ratio of the magnetic particles, $\mu_{i}$ is the magnetic permeability of the magnetic particles, μ is the magnetic permeability of the base liquid, a is the size of the magnetic particles, r is the distance from the center of the magnetic particles, and θ is the angle between the direction of the magnetic field and the line connecting the spherical centers of the magnetic particles.

According to Equation (1), in order to make the MPF have a large shear yield stress, the magnetic particles need to have a high content, a large size, and high magnetic permeability. However, the high content of magnetic particles will lead to a sharp increase in the viscosity of the MPF, affecting the fluidity, which is not conducive to the renewal of MPF, and then affect the material removal efficiency. The excessive size of magnetic particles will affect the dispersion stability of MPF and may lead to particle agglomeration. Therefore, the content and size of the magnetic particles must be selected appropriately. In this study, micron-sized CIPs (MCIP-HD-R-3, Shaanxi Xuliheng New Materials Co., Ltd., Baoji, China) were temporarily selected as the magnetic particles, which have the advantages of high purity, high magnetic permeability, and low coercivity.

#### 2.1.2. Influence of Abrasive Particles

**Figure 1:**
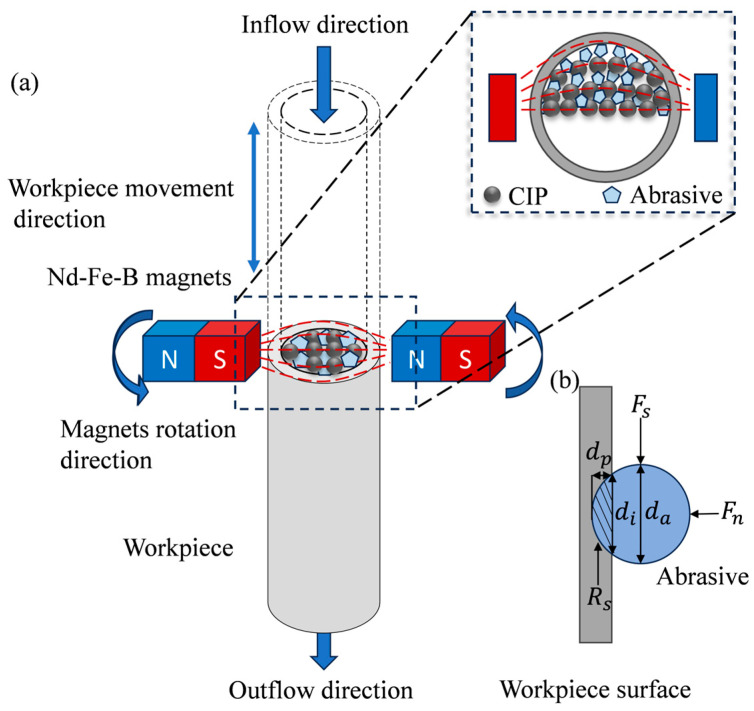
Schematic diagram: (**a**) principle of RMRF—the polishing liquid flows inside the workpiece, and the magnetic field moves helically with respect to it; (**b**) processing forces acting on a single abrasive.

As the component of an MPF that directly engages the workpiece surface, abrasive particles critically determine material removal performance, content, size, and hardness, and morphological characteristics exert a direct influence on removal capability. The force applied to a single abrasive particle in the polishing process is shown in the Figure 1b; under the action of pressure $F_{n}$, the abrasive particle is pressed into the surface of the workpiece to a certain depth $d_{p}$. Under the application of shear force $F_{s}$, the abrasive particle undergoes relative motion with respect to the workpiece surface to achieve material removal. The pressure $F_{n}$ and the shear force $F_{s}$ mainly come from the magnetic chain structures formed by the magnetic particles under the magnetic field. The material removal behavior can only occur when the shear force $F_{s}$ applied to the abrasive particle is greater than the resistance $R_{s}$ from the material, and the formulae for the resistance $R_{s}$ are as follows [30]:

(4) $$R_{s}=A_{p}\sigma$$

(5) $$\frac{1}{4}{d_{i}}^{2}=\left(d_{a}-d_{p}\right)d_{p}=d_{a}d_{p}$$

(6) $${d_{i}}^{2}=\frac{F_{n}}{C_{v}H_{v}}$$

(7) $$A_{p}=\frac{d_{a}^{2}}{4}\sin^{-1}\frac{d_{i}}{d_{a}}-\frac{1}{2}d_{i}\left(\frac{d_{a}}{2}-d_{p}\right)={d_{a}}^{\frac{1}{2}}{d_{p}}^{\frac{3}{2}}={d_{a}}^{\frac{1}{2}}{\left(\frac{F_{n}}{C_{v}H_{v}}\right)}^{\frac{3}{2}}$$

σ is the yield strength of the material being processed; $A_{p}$ is the cross-sectional area of the indentation between the abrasive particle and the surface; $C_{v}$ is the coefficient related to Vickers hardness; $H_{v}$ is the Vickers hardness of the material being processed.

The volume of material cut by a single abrasive particle per unit time can be expressed as

(8) $$dV=A_{p}dL={d_{a}}^{\frac{1}{2}}{d_{p}}^{\frac{3}{2}}dL={d_{a}}^{\frac{1}{2}}{\left(\frac{F_{n}}{{4C}_{v}H_{v}}\right)}^{\frac{3}{2}}dL$$

dL is the cutting length of the abrasive particle per unit of time.

Equations (4), (6) and (8), indicate that increasing abrasive size enhances the volumetric removal rate and overall material removal efficiency. However, this also requires a greater shear force from the magnetic chain structure, thereby demanding a higher shear yield strength of the MPF.

Diamond, alumina, silicon carbide, and cerium oxide are usually used as abrasive particles in MPF. Among them, diamond particles have the highest hardness and the highest material removal efficiency but are also the most expensive. Cerium oxide has low hardness and is commonly used to process optical components. Referring to relevant research and basing on previous processing experiments, micron alumina abrasive (ALP, Zhejiang Lixie Instrument & Equipment Co., Ltd., Yiwu, China) particles were selected in this study. Alumina abrasive particles possess an optimal hardness that affords sufficient material removal capability while minimizing the risk of inducing deep scratches or subsurface damage on the workpiece surface. Increasing the content of abrasive particles in the MPF increases the number of particles engaged in polishing per unit volume. However, excessive particle loading may impede the formation of the magnetic chain structure, thereby adversely affecting the shear yield strength of the fluid. The content and size of magnetic particles and abrasive particles in the MPF used for polishing the inner surface of slender tubes need to be investigated experimentally.

Dispersants are essential components in magnetorheological polishing fluid, playing a crucial role in the stable dispersion of solid particles within the polishing fluid. Commonly used dispersants include hydroxypropyl methylcellulose, sodium polystyrene sulfonate, and sepiolite [31,32]. In this study, the dispersion stabilizer was hydroxypropyl methylcellulose, and the base fluid was deionized water.

#### 2.1.3. Polishing Principle

Figure 1a illustrates the principle of the RMRF method for the inner surface of slender tubes. Two bar magnets are radially arranged along the tube, with opposite polarities near the wall side (N pole facing S pole). The workpiece is vertically positioned between the two magnets, maintaining a fixed gap relative to the magnets.

In the polishing process, the workpiece reciprocates at a specified speed and stroke while the magnets rotate counterclockwise around the workpiece axis at a predetermined rate. Under externally applied pressure, the MPF flows inside the workpiece and is continuously renewed. Within the workpiece, the CIPs in the polishing fluid align along the magnetic induction lines to form chains. Driven by the combined effects of the workpiece’s reciprocating motion, the rotation of the magnets, and the flow of the polishing fluid, these chains move in a helical trajectory relative to the inner surface. Additionally, due to the magnetic field gradient, the polishing fluid concentrates toward the inner wall, generating radial pressure. This causes the abrasive particles to press against the inner surface of the workpiece under the influence of the chains and produce relative motion with respect to the inner surface, thereby achieving the removal of the material.

### 2.2. Model Building of Simulation

Based on the polishing principle proposed in Section 2.1, we established a simulation model under multiple physical fields to simulate the polishing process, providing theoretical guidance for the design of processing apparatus and subsequent experimental verification, and preliminarily verifying the feasibility of the proposed polishing principle. The magnetic field distribution and the rheological characteristics of the MPF are the primary factors governing its processing capability. It is imperative that the rotating magnetic field establish a sufficiently substantial magnetic field gradient within the tube to drive fluid motion effectively. The material removal performance of the MPF is principally determined by the magnitude of shear stress applied to the workpiece surface. Accordingly, a preliminary transient analysis of the process is required to elucidate the influence of magnetic field distribution, shear stress, and body force on material removal.

To simulate the polishing process in COMSOL Multiphysics 6.2, an accurate mathematical model must be established to characterize the rheological properties of the MPF. Commercial MPF prepared by Chongqing Institute of Materials was selected for simulation. The basic parameters of the water-based MPF used in the simulation are summarized in Table 1. Although the Bingham model is widely used to describe the mechanical behavior of such fluids, it fails to adequately capture shear-thinning phenomena and exhibits a discontinuity in shear stress as the shear rate approaches zero [33], which can compromise simulation accuracy. Consequently, the Bingham–Papanastasiou model was adopted to describe the rheological behavior of the MPF, with its constitutive relationship presented as follows:

(9) $$\tau =\mu_{p}\dot{\gamma }+\tau_{y}\left[1-\exp\left(-m_{p}\dot{\gamma }\right)\right]$$

where $\mu_{p}$ is the plastic viscosity, $\dot{\gamma }$ is the shear rate, $\tau_{y}$ is the yield stress, $m_{p}$ is a model parameter, and $\tau_{y}$ and $m_{p}$ are rheological parameters, which change with magnetic induction intensity.

**Figure 2:**
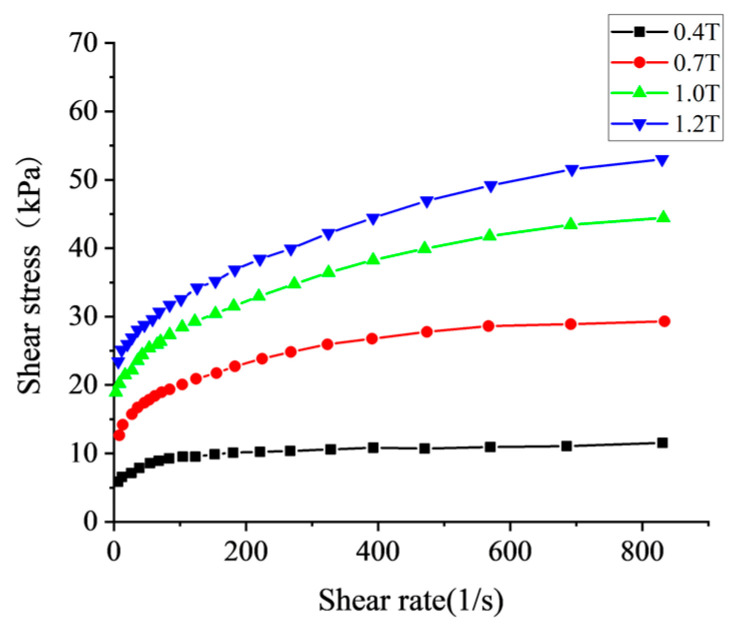
The flow curves of magnetorheological polishing liquid under different magnetic field intensities.

Flow curves of the MPF were measured with a rotational rheometer under varying magnetic field intensities, as shown in Figure 2.

By constructing an error function for the shear stress and applying optimization theory, the relationship between each parameter and magnetic induction intensity B was calculated as follows [34]:

(10) $$\tau_{y}=-2.452\times 10^{4}\times B^{3}+5.892\times 10^{4}\times B^{2}+11250\times B$$

(11) $$m_{p}=0.02536\times B^{2}-0.05333\times B+0.03453$$

The impact of magnetic field on the flow state of MPF can be quantified by the volume force $F_{v}$ acting on the polishing fluid, through which the physical field coupling between magnetic field and flow field can be realized in COMSOL Multiphysics. The volume force $F_{v}$ can be expressed as

(12) $$F_{v}=M\cdot \nabla B_{0}=\mu_{0}M\cdot \nabla H$$

where M is the magnetization vector, $B_{0}$ is magnetic induction vector, $\mu_{0}$ is the permeability of the vacuum, and H is magnetic field intensity.

**Figure 3:**
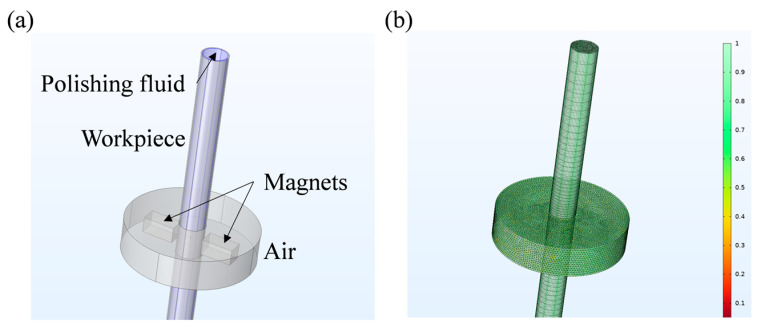
Models in COMSOL: (**a**) four essential domains; (**b**) high-quality mesh.

Based on the polishing principle proposed in Section 2.1, a simplified model for polishing the inner surface of the tube was developed using COMSOL Multiphysics, as depicted in Figure 3. To optimize computational efficiency while maintaining simulation accuracy, the model size was minimized by retaining only four essential domains, the workpiece, polishing fluid, magnet, and air, as shown in Figure 3a. The first three are the main parts directly related to the processing, and the air ensures the correct conduction of the magnetic field near the magnets.

The workpiece is modeled as a cylindrical tube with an inner diameter of 9 mm, a length of 150 mm, and a wall thickness of 0.5 mm, which is consistent with the dimensions of the actual processed workpiece. During the actual processing, the magnetic field performs rotational motion and up-and-down reciprocating motion relative to the workpiece. As the focus of the simulation is on the processing process within the local area, only the rotational motion of the magnetic field is retained.

To validate the feasibility of the polishing principle described in Section 2.1, numerical simulations of the process were conducted. To ensure the accuracy of the computational results, under the coupled magnetic and flow field conditions, the transient analysis of the polishing process under different inlet pressures and magnetic field rotational speeds was carried out by setting parametric scanning. The computational domain was meshed using mapped and swept techniques, yielding a high-quality mesh with an average quality factor of 0.686, as shown in Figure 3b. Additionally, the arbitrary Lagrange–Euler (ALE) method was employed to define dynamic meshing for the rotating regions of the magnets and air domains. For the material settings, air, stainless steel workpiece, and permanent magnet can be directly found and applied in the material library of the simulation software. For MPF, it needs to be set according to the mathematical formula calculated in Section 2.2 and the Bingham–Papanastasiou model.

### 2.3. Experimental Methods

**Figure 4:**
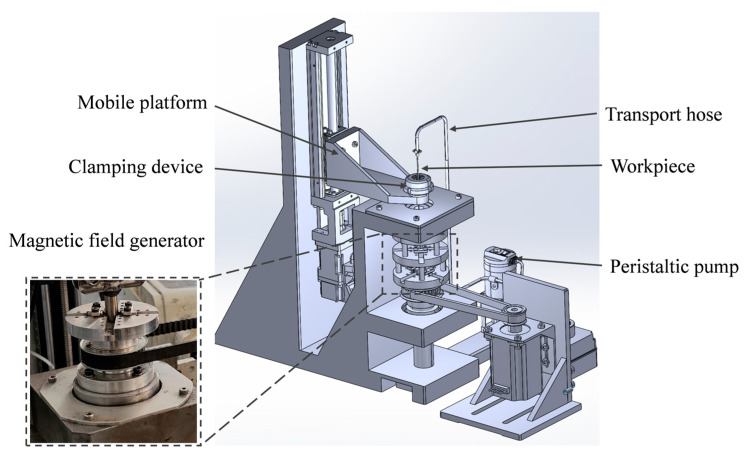
Processing apparatus.

Based on the polishing principle in Section 2.1, the processing apparatus was designed and constructed, as illustrated in Figure 4. The apparatus primarily consists of a rotating magnetic field module, a workpiece motion module, a polishing fluid delivery module, a polishing fluid stirring device, and a suite of ancillary components. Specifically, a pair of bar magnets are mounted on a rotating platform and driven by a motor through a belt to generate a magnetic field that rotates around the axis of the workpiece. The workpiece is mounted on a displacement stage with *z*-axis translation capability, enabling reciprocating vertical motion. To facilitate cleaning of the MPF delivery system after processing, two peristaltic pumps and hoses are utilized to continuously circulate the polishing fluid. During processing, the polishing fluid is continuously stirred in a mixing drum to ensure uniform dispersion.

Based on the preliminary experimental results, the processing parameters outlined in Table 2 have been initially determined to achieve effective material removal from the inner surface of the workpiece. The workpiece to be processed is a hollow circular pipe with an outer diameter of 10 mm, an inner diameter of 9 mm, and a length of 200 mm, and its material is 316 L stainless steel.

The MPF is a critical component of the magnetorheological finishing process, as it directly influences efficiency of the processing and the final quality. The polishing fluid was prepared as follows: first, the hydroxypropyl methylcellulose is gradually added to deionized water and stirred continuously for 30 min to form a stable continuous phase. Simultaneously, the CIPs and alumina polishing particles are mixed to create a dispersed phase. Subsequently, with continuous stirring maintained, the dispersed phase is gradually incorporated into the continuous phase. Finally, the mixture is stirred at 500 rpm for 1 h using a mechanical stirrer to obtain a uniformly mixed MPF.

This polishing fluid behaves as a Bingham fluid, rapidly transitioning to a quasi-solid state when subjected to a magnetic field. This transition results in a dramatic increase in viscosity of the MPF and a significant reduction in flowability within the region affected by the magnetic field. Therefore, it is essential to ensure that the polishing fluid is fully circulated through the workpiece, hoses, and stirring drum before installing the magnets on the rotating platform and subsequently activating both the magnetic field rotation and the workpiece motion devices. Additionally, due to the inevitable evaporation of water during circulation, which diminishes the flowability of the polishing fluid and alters its properties, the base fluid must be replenished in accordance with the evaporation rate throughout the processing.

In this study, a series of polishing experiments were conducted to systematically investigate the effects of various component parameters of the MPF, including the volume contents and the sizes of the solid particles (CIPs and abrasives), on the surface roughness of the machined workpiece. Unless otherwise specified, all contents and concentrations are expressed in vol.%. The objective was to optimize the formulation of the polishing fluid to achieve a uniform finish on the inner surface of the slender tube. The parameters and their corresponding value ranges are presented in Table 3. Processing time is also an important factor affecting processing quality: If the processing time is not long enough, the original defects on the surface of the workpiece cannot be adequately removed. If the processing time is too long, the smooth surface that has been processed may be scratched to generate new defects, resulting in a renewed surface quality deterioration. Therefore, it is necessary to analyze the surface characteristics at different processing stages in order to determine the appropriate processing time.

Since the area to be processed is the inner surface of a slender tube, in situ measurements cannot be conducted without damaging the workpiece. Therefore, the inner surface roughness can only be measured by utilizing a wire cutting machine to section the workpiece. The measurement procedure in this study is as follows: after processing, the workpiece is sequentially immersed in acetone and anhydrous ethanol for ultrasonic cleaning for 30 min each. Subsequently, the workpiece is cut using a wire cutting machine. The inner surface roughness is measured with a ZYGO white light interferometer (NewView 9000, Zygo CO., LTD., Middlefield, CT, USA), with 3 to 5 equally spaced measurement points taken along the axial direction on both sides of the inner surface, and their average value is calculated. Additionally, the morphology of the processed inner surface is examined using a VHX-1000 super deep field microscope (KEYENCE (CHINA) CO., LTD., Shanghai, China).

## 3. Results

### 3.1. Simulation Results

**Figure 5:**
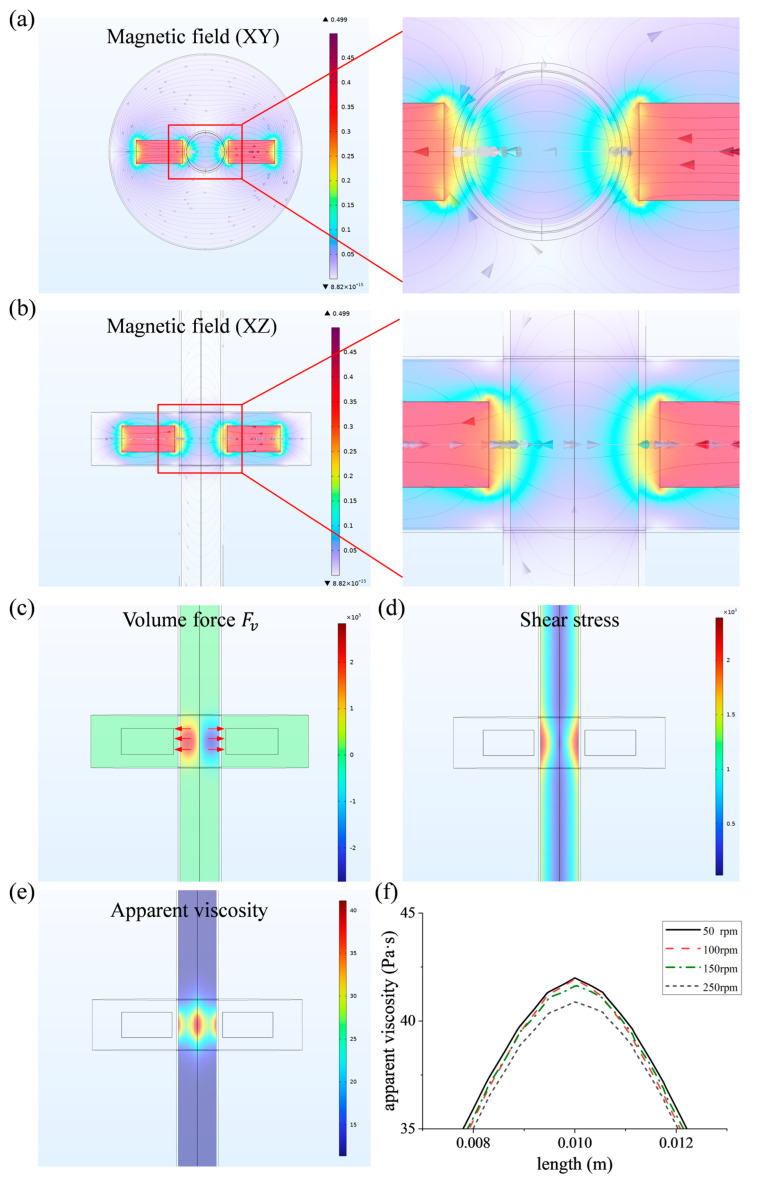
Simulation result: (**a**) distribution of magnetic field (XY plane); (**b**) distribution of magnetic field (XZ plane); (**c**) distribution of the volume force $F_{v}$; (**d**) distribution of the shear stress; (**e**) distribution of the apparent viscosity; (**f**) variation of apparent viscosity along the workpiece axis at different rotational speeds.

The distribution of the magnetic field significantly influences material removal behavior during magnetorheological polishing. Since the rotational speed of the magnetic field primarily affects the action period, the rotation speed of 150 rpm is taken as an example to analyze the magnetic field distribution. To enhance the effect of magnetic field near the inner surface of the workpiece, the gap between the magnets and the wall of the workpiece was set to 1 mm. Figure 5a,b display the distribution of magnetic field, which reveal that the majority of the magnetic induction lines penetrate the workpiece wall perpendicularly. The highest magnetic induction density is observed in the region of the inner surface opposite the magnet center, approximately 0.23 T, and the magnetic induction density along the workpiece axis is only 0.06 T. This magnetic field distribution exhibits a pronounced gradient variation, which not only enhances the shear yield strength of the MPF in the surface area to be processed but also promotes effective fluid flow and renewal in the central region of the workpiece.

Material removal relies on the interaction forces between the abrasive particles and the workpiece surface, with both the distribution and magnitude of these forces significantly impacting surface quality and processing efficiency. Figure 5c illustrates the distribution of the volume force $F_{v}$ along the *x*-axis, indicating that the force direction aligns with the direction of magnetic field and is primarily concentrated in the region opposite the center of the magnets. This distribution corresponds well with the distribution of magnetic induction intensity, which is beneficial to control the interaction force between the MPF and the workpiece by controlling the magnetic field. Figure 5d illustrates that the shear stress near the inner surface of the workpiece is considerably higher than that in the central region, reaching a maximum at the workpiece wall and varying with the rotation of the magnetic field along the circumferential direction of the workpiece. This distribution helps to achieve material removal.

Under the influence of the magnetic field, the MPF exhibits pronounced rheological behavior, which is critical for achieving material removal. Apparent viscosity is a critical parameter for evaluating the properties of the MPF. Although increased apparent viscosity enhances the cutting force of abrasive particles, an excessively high viscosity restricts fluid flow and impedes particle renewal, thereby undermining processing quality.

Conversely, if the apparent viscosity is too low, the abrasive particles may not effectively remove surface defects, resulting in reduced polishing efficiency. Figure 5e,f show the distribution of the apparent viscosity and its variation along the workpiece axis at different rotational speeds. It can be found that the apparent viscosity rises significantly in the magnetic field range and its magnitude is also related to the rotational speed. Specifically, the apparent viscosity decreases with the increase in rotational speed, which may be related to the process of magnetic chain formation. At high rotational speeds, the chain structure formed by the magnetic particles is destructed, which in turn decreases the viscosity and shear yield strength of the MPF, showing characteristics of shear thinning and weakening the control of the abrasive particles [35]. However, on the other hand, with the increase in magnetic field rotational speed, the abrasive particles can remove the material at a higher frequency, and the number of polishing cycles per unit time increases, which in turn increases the polishing efficiency. Therefore, considering the effect of the magnetic field rotational speed comprehensively, the magnetic field rotational speed in the subsequent processing experiments is temporarily set at 150 rpm.

### 3.2. Experiment Results

The MPF is the critical component of the magnetorheological polishing technique. A series of experiments were designed and carried out to figure out the key influence factors on the surface quality, on the basis of which the composition of the MPF was optimized. Under the conditions of the optimal formulation, the relationship between surface roughness and processing time was studied. The processing parameters were kept consistent, as outlined in Table 2.

#### 3.2.1. Influence of Volume Content of CIPs on Surface Roughness

**Figure 6:**
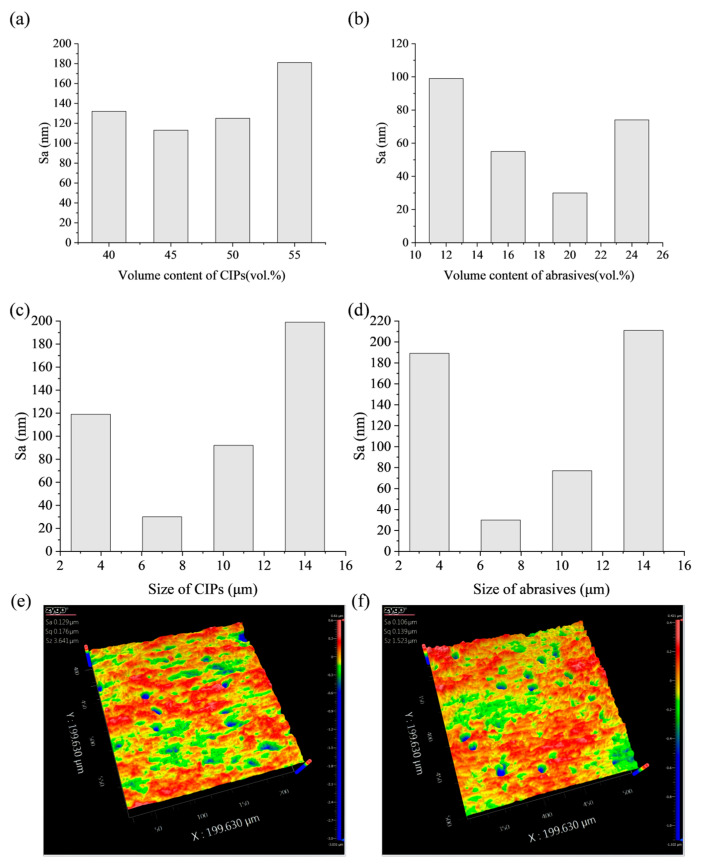
Experiment results: (**a**) relationship between surface roughness and volume content of CIPs; (**b**) relationship between surface roughness and volume content of abrasives; (**c**) relationship between surface roughness and size of CIPs; (**d**) relationship between surface roughness and size of abrasives; (**e**) white light interference image of CIPs with large particle size; (**f**) white light interference image of abrasives with small particle size.

To investigate the effect of the volume content of CIPs on the surface roughness after processing, the volume content of CIPs was varied according to the values presented in Table 3, whereas the volume contents of alumina abrasive and hydroxypropyl methylcellulose were fixed at 9% and 1%, respectively. Additionally, the average sizes of both the CIPs and alumina abrasives were maintained at 7 μm. The results are shown in Figure 6a, indicate that as the volume content of CIPs increases, the internal surface roughness shows a tendency of decreasing and then increasing. The optimal value of CIP volume content is 45%, which indicates that excessively low or high CIP content is not favorable to the improvement in processing capability.

**Figure 7:**
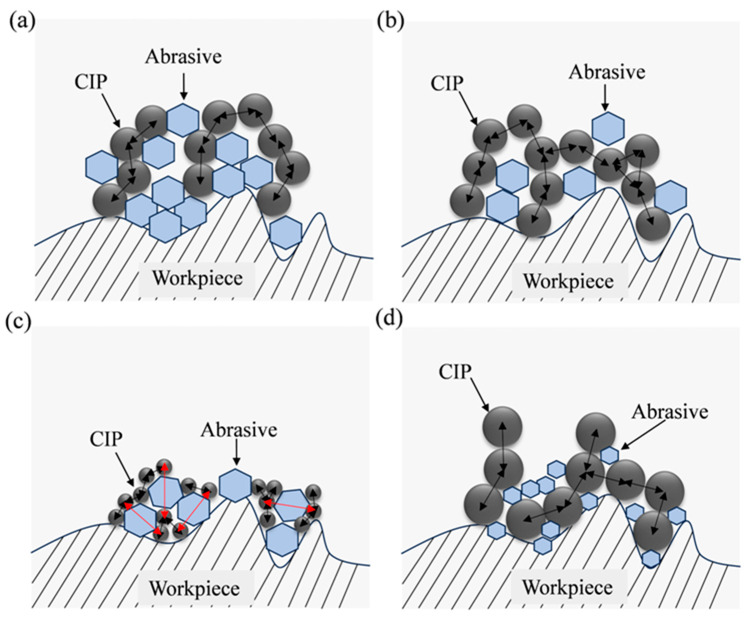
Schematic diagram: (**a**) when the content of CIPs is too low or the content of abrasive is too high; (**b**) when the content of CIPs is too high or the content of abrasive is too low; (**c**) when the size of CIPs is too small or the size of abrasives is too large; (**d**) when the size of CIPs is too large or the size of abrasives is too small.

As the content of CIP decreases, the number of magnetic particles in the MPF reduces, resulting in a decrease in the chain structure formed under the action of the magnetic field, which leads to a decline in abrasive particle control. According to Equations (1)–(8), as the number of magnetic particles decreases, the shear yield strength of the MPF decreases, and the polishing pressure provided by CIPs to the abrasive particles is insufficient, resulting in a smaller cutting depth of the abrasive particles on the workpiece surface, as shown in Figure 7a. Furthermore, the material removal efficiency decreases, and surface defects cannot be fully removed. In contrast, a high content of CIPs results in elevated fluid viscosity, thereby impeding the relative movement between the abrasive particles and the inner surface of the workpiece. Measurements taken with a rotational rheometer indicate that when the volume content of CIPs exceeds 55%, the viscosity surpasses 20,000 mPa·s (at a shear rate of 10 s^−1^), resulting in greatly reduced flowability and decreased processing efficiency, which ultimately leads to inferior polishing capability.

#### 3.2.2. Influence of Volume Content of Abrasive Particles on Surface Roughness

During processing, abrasive particles directly interact with the workpiece surface and play a critical role in material removal. To investigate the effect of the volume content of abrasive particles on surface roughness after processing, the volume content of abrasive particles was varied according to the values shown in Table 3, whereas the volume contents of CIPs and hydroxypropyl methylcellulose were fixed at 45% and 1%, respectively. Additionally, the average sizes of both the CIPs and alumina abrasives were kept consistent at 7 μm. Figure 6b illustrates the relationship between the surface roughness of the inner surface of the machined workpiece and the volume content of abrasive particles. When the volume content of abrasive particles is less than 20%, the surface roughness value decreases gradually with the increase in volume content of abrasive particles and the lowest value is obtained at 20%. If the content of abrasive particles is further increased, the surface roughness value will rise significantly.

When the content of abrasives is too low, the number of abrasive particles available per unit volume in the MPF is insufficient for effective cutting. This limitation restricts material removal efficiency and prevents the timely elimination of initial defects of the workpiece, as shown in Figure 7b. As the content of abrasives increases, a greater number of abrasive particles engage in the cutting process, enhancing material removal efficiency and promoting a more uniform distribution of abrasives within the chain structures. This improved distribution minimizes direct contact between magnetic particles and the workpiece surface, alleviating local stress concentrations and thereby improving surface quality. However, if the content of abrasives becomes excessively high, the rheological equilibrium of the MPF is disrupted. The surplus abrasive particles hinder the formation of magnetic chain structures in the magnetic field, leading to a non-uniform distribution and agglomeration of abrasive particles, as shown in Figure 7a. This can result in deep scratches and secondary defects on the processed surface, ultimately degrading surface quality.

#### 3.2.3. Influence of CIP Size on Surface Roughness

To investigate the effect of the size of CIPs on surface roughness after processing, this study varied the size of CIPs based on the values presented in Table 3. The volume content of CIPs was maintained at 45%, while the volume contents of alumina abrasives and hydroxypropyl methylcellulose were kept at 20% and 1%, respectively.

The experimental results are presented in Figure 6c: optimal processing performance is attained at a CIPs size of 7 µm; deviations above or below this size result in increased surface roughness value. At this size, the CIPs can rapidly respond to the dynamic magnetic field, forming magnetic chain structures due to their high surface-to-volume ratio. Furthermore, the high surface energy of the CIPs increases the synergistic effect with the abrasive particles, thereby improving the polishing performance. However, for CIP sizes below this threshold, although the particles rapidly respond to the magnetic field and form magnetic chain structures, those chains exhibit reduced strength and are more prone to fracture, consistent with Equations (1)–(3). Smaller CIP particles diminish interparticle forces and thereby lower the shear yield strength of the MPF. This leads to a weakening of radial pressure on the abrasive particles, resulting in a reduction in the depth of cut of the abrasive particles and precluding the generation of sufficient shear stress on the surface of the workpiece, as shown in Figure 7c, ultimately affecting the material removal efficiency and hindering the elimination of deeper defects. Furthermore, as the size of CIPs decreases, the specific surface energy increases, thereby promoting particle agglomeration and consequently compromising the uniformity of processing.

Conversely, increasing CIP size elevates the shear yield strength of the polishing fluid, but produces coarser magnetic chain structures with reduced compliance to curved surfaces, which is not conducive to the full adhesion of the “flexible polishing brush” to the workpiece surface. In addition, too large a size promotes direct contact between the magnetic particles and the surface to be processed, resulting in the inaccessibility of the abrasive particles to cut effectively against the workpiece surface, as shown in Figure 7d. Due to being subjected to greater radial pressure, abrasive particles are more likely to be embedded in the surface of the workpiece causing craters, as shown in the white light image in Figure 6e.

#### 3.2.4. Influence of Abrasives Size on Surface Roughness

As a key component of MPFs directly involved in material removal, the size of abrasive particles directly affects the cutting behavior. To investigate the effect of the size of abrasive particles on surface roughness after processing, this study varied the size of abrasive particles based on the values presented in Table 3. The volume content of CIPs was maintained at 45%, while the volume contents of alumina abrasives and hydroxypropyl methylcellulose were kept at 20% and 1%, respectively.

The experimental results are presented in Figure 6d. The results indicate that when the abrasive size decreases, the depth of cut increases according to Equations (5) and (6). The abrasive particles are more likely to be embedded in the surface of the workpiece, and produce round-hole defects, which is similar to the situation where the size of the CIPs is too large, as shown in Figure 6f. In addition, when the size of the abrasive particles is small, a single abrasive particle removes less volume of material per unit of time, resulting in lower cutting efficiency and poor processing results. On the contrary, when the size of the abrasive particles increases, the radial pressure generated by the magnetic particles is not enough for the abrasive particles to generate a sufficient depth of cut, and micro-plowing occurs mainly. In addition, according to Equations (1)–(3), an increase in the size of the abrasive particles results in an increase in the distance between the magnetic particles, as shown in Figure 7c, which in turn results in a decrease in the shear yield strength and a further decrease in the material removal ability.

Only when the abrasive size the same as that of the magnetic particles, that is, both are 7 μm, the abrasive particles not only have an appropriate cutting depth but can also be effectively controlled by the magnetic chains, thereby achieving controllable micro-cutting and effective removal of surface defects. In this case, the magnetic field response frequencies of the abrasive particles and magnetic particles are similar, leading to the formation of a stable “magnetic chain-abrasive” structure through coupling effects, and finally realizing the uniform polishing of the workpiece surface. In summary, under the processing conditions of this study, the best processing result is achieved when both the abrasive and CIP sizes are 7 μm. This conclusion is consistent with the findings of Das et al. [36] that the size of abrasive particles should be close to the magnetic particles in a rotating magnetorheological process.

#### 3.2.5. Influence of Processing Time on the Surface Roughness

**Figure 8:**
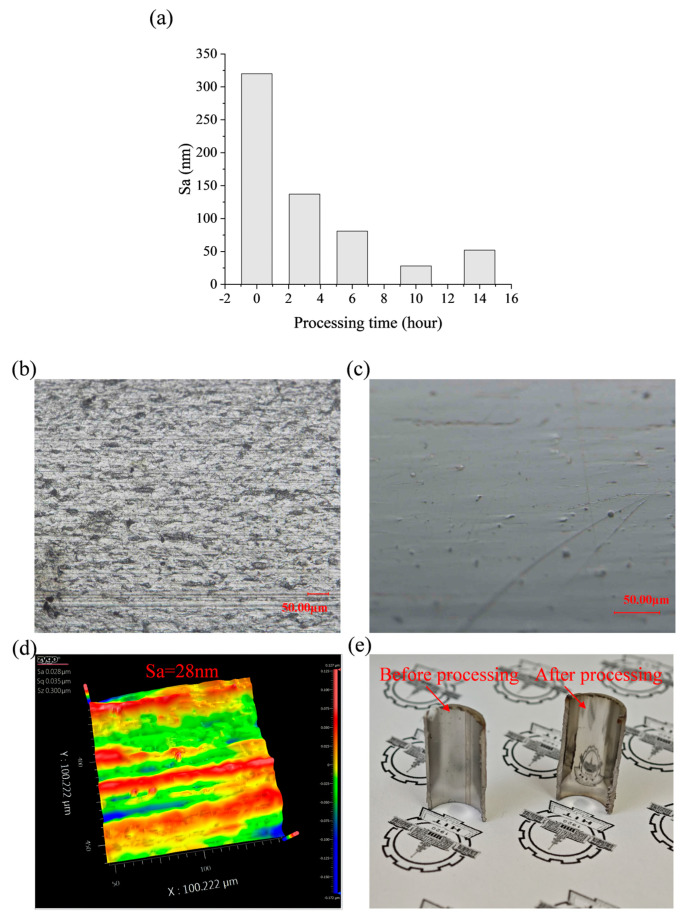
(**a**) Relationship between surface roughness and processing time; (**b**) microscope image before processing; (**c**) microscope image after processing; (**d**) white light interference image after processing; (**e**) image of the actual workpiece before and after processing.

Based on the findings from Section 3.2.1, Section 3.2.2, Section 3.2.3 and Section 3.2.4, the optimal formulation of the MPF was determined to be 45 vol% CIPs, 20 vol% alumina abrasives, 1 vol% hydroxypropyl methylcellulose, and 34 vol% deionized water, with average sizes of both the CIPs and the alumina abrasives of 7 μm. In the magnetorheological polishing process, excessively long processing time may lead to over-polishing of the surface, thereby compromising surface quality. To investigate the effect of processing time on surface roughness and surface morphology, experiments were conducted using the optimal formulation under the processing parameters shown in Table 2 for durations of 3, 6, 10, and 14 h. The relationship between surface roughness and processing time is shown in Figure 8a.

The results indicate that after 3 h of processing, the surface roughness decreases rapidly to 137 nm, and most of the initial surface defects are removed; then, the reduction rate of roughness gradually slows down. After 10 h of processing, the surface roughness reaches a minimum value of 28 nm. The super-depth-of-field image is shown in Figure 8c, and the surface defects are basically removed; however, by continuing the processing, the surface roughness increases to 52 nm instead.

In the initial stages of processing, there are large-scale defects on the surface of the workpiece. These defects can be rapidly eliminated by sharp abrasive particles within a short time, resulting in a swift decrease in the value of surface roughness. As processing continues, the size and number of defects to be removed gradually decrease, leading to a flatter surface. This flattening reduces the pressure per unit area at the contact region between the abrasive particles and the workpiece, ultimately resulting in a decrease in the rate of material removal. Furthermore, during the early stages of processing, the abrasive particles are sharp, contributing to high cutting efficiency. However, as processing time extends, the cutting edges of the abrasive particles become worn, which slows the reduction in roughness during the later stages. After 10 h of processing, the surface roughness reaches its minimum value. If processing continues beyond this point, it leads to over-polishing.

Considering these factors, under the current formulation of MPF and the established processing parameters, the optimal surface quality can be achieved after 10 h of processing. This results in a reduction in surface roughness Sa to 28 nm and a decrease in Sz from 10.972 μm to 0.3 μm. The microscope images taken before and after processing and the white light interference images after processing are presented in Figure 8b–d. Compared to the initial surface, the surface defects of the workpiece were effectively eliminated following processing; the actual workpiece image is displayed in Figure 8e, and the surface clearly reflects the image. The feasibility of the proposed processing method has been demonstrated.

## 4. Conclusions

This paper presents a novel rotary magnetorheological polishing method for the inner surfaces of slender tubes. A multiphysics simulation of the magnetorheological polishing process was conducted using COMSOL, the composition of MPF was optimized through single-variable experiments, and the appropriate processing time was determined. The following conclusions can be drawn:

1. Transient and steady-state analyses of the magnetorheological polishing process were conducted under the multiphysics coupling of magnetic and flow fields to investigate the influence of various factors, including magnetic field strength, body force under the magnetic field, shear stress, and viscosity, on the material removal process. The force generated under the magnetic field is primarily concentrated in the region opposite the center of the magnets and has good synchronization with the movement of the magnetic field, thereby allowing for precise control of the material removal behavior of the MPF on the workpiece surface by controlling the magnetic field distribution.
2. The feasibility of the proposed polishing method was validated through processing experiments, the relationship between surface roughness and various parameters of the MPF was investigated using single-variable experiments, and the composition of the magnetorheological polishing solution was optimized. The optimal formulation consisted of 45 vol% CIPs, 20 vol% alumina abrasives, 1 vol% hydroxypropyl methylcellulose, and 34 vol% deionized water, with the optimum average sizes for both the CIPs and the alumina abrasives being 7 μm.
3. Utilizing the optimum formulation, the effect of processing time on surface roughness was investigated. Under the optimum time, the internal surface roughness of the 316L stainless steel slender tube workpiece was reduced from Sa 320 nm to Sa 28 nm, and the Sz was reduced from 10.972 μm to 0.3 μm, which effectively eliminated the initial surface defects and obtained a flat and smooth surface.

## Figures and Tables

**Table 1:** Basic parameters of MPF.

Parameters	Value
Density	2.7 g/m^3^
Relative magnetic permeability	2.63
Plasticity viscosity	11.34 Pa∙s

**Table 2:** Processing parameters.

Parameters	Value
Workpiece	D = 10 mm, d = 9 mm, L = 150 mm 316 L stainless steel
Bar magnet	6 mm × 6 mm × 12 mm Nd-Fe-B, Br = 0.5 T
Magnetic field intensity of machined surface	0.25 T
Rotation speed of the magnetic field	150 rpm
Distance of workpiece movement	20 mm
Speed of workpiece movement	1.1 mm/s
Processing time	10 h

**Table 3:** Parameters and value ranges of MRF.

Parameters	Value
Volume content of CIPs (vol.%)	40, 45, 50, 55
Volume content of abrasives (vol.%)	12, 16, 20, 24
Volume content of hydroxypropyl methylcellulose (vol.%)	1
Size of CIPs (μm)	3.5, 7, 10.5, 14
Size of abrasives (μm)	3.5, 7, 10.5, 14

## Data Availability

The original contributions presented in this study are included in the article. Further inquiries can be directed to the corresponding author.
